# STING signaling is a potential immunotherapeutic target in colorectal cancer

**DOI:** 10.7150/jca.32806

**Published:** 2019-08-27

**Authors:** Hong Jae Chon, Hyojoong Kim, Jung Hyun Noh, Hannah Yang, Won Suk Lee, So Jung Kong, Seung Jun Lee, Yu Seong Lee, Woo Ram Kim, Joo Hang Kim, Gwangil Kim, Chan Kim

**Affiliations:** 1 Medical Oncology, CHA Bundang Medical Center, Seongnam, Korea; 2 Laboratory of Translational Immuno-Oncology, Seongnam, Korea; 3 Department of Surgery, CHA Bundang Medical Center, Seongnam, Korea; 4 Department of Pathology, CHA Bundang Medical Center, Seongnam, Korea; 5 CHA Medical School, CHA University, Seongnam, Korea

**Keywords:** STING, Colorectal cancer, Prognosis, Immunotherapy, Inflamed tumor

## Abstract

**Background**: Stimulator of Interferon Genes (STING) is an innate immune sensor for cytosolic DNA. STING signaling activation is indispensable for type I interferon response and the anti-cancer immune response by CD8^+^ T cells. The aim of this study was to characterize intratumoral STING expression pattern and its clinical implication in colorectal cancer (CRC).

**Methods**: We analyzed STING and CD8 expression in 225 CRC patients who underwent surgical resection. Clinicopathological variables and survival outcomes were analyzed according to STING expression levels. Mice with syngeneic MC38 tumors were also treated with a STING agonist, and tumor microenvironments were analyzed using immunofluorescent staining and flow cytometry.

**Results**: Distinct STING expression was observed in the CRC tumor specimens. Patients with higher STING expression had early stage cancer with increased intratumoral CD8^+^ T cell infiltration and less frequent lymphovascular invasion. Compared to CRC patients with lower STING expression, those with higher STING expression had longer overall and recurrence-free survival. Multivariate Cox regression model also revealed higher STING expression to be an independent prognostic factor for better overall survival. When MC38 colon tumors were treated with intratumoral injection of STING agonist, tumor growth was remarkably suppressed with increased intratumoral CD8^+^ T cell infiltration. Moreover, T-cell activation markers, ICOS and IFN-γ, were also upregulated in CD8^+^ T cells, indicating enhanced effector T cell function after STING treatment.

**Conclusion**: We confirmed the distinct STING expression in CRC and demonstrated its independent prognostic value in survival outcomes. STING could be a potential therapeutic target that enhances anti-cancer immune response in CRC.

## Introduction

Colorectal cancer (CRC) remains the third most common cancer worldwide, as over two million patients were newly diagnosed annually and more than one million died of CRC 1, 2. Although the rates of CRC death are dropping in recent decades, the 5-year survival rate was only 13% in stage IV CRCs. Therefore, a CRC is a life-threatening malignancy with a high demand for effective treatment, especially when diagnosed at an advanced stage 3. Over the last decade, advances in systemic chemotherapy and the introduction of the 'continuum of care' strategy have made remarkable progress on CRC treatment. However, the biologic heterogeneity of CRC among patients still results in discrepancies in treatment response and survival outcome, which makes it harder to treat CRC 4, 5.

The immune system is essential for detecting and eliminating cancer cells, and adaptive anti-cancer immune responses driven by effector T cells are especially indispensable in the immune surveillance of cancer 6-8. Since this immunologic monitoring is defective in many human malignancies, immunotherapeutic agents that can potently augment effector T cell function against cancer are being developed and actively introduced into clinical practice recently 7, 9. However, the therapeutic efficacy of cancer immunotherapy in CRC is severely hampered due to the poorly-immunogenic tumor cells and immunosuppressive tumor microenvironment 10-12. Therefore, a better understanding of the immunologic features of CRC and identification of novel immune targets are necessary to overcome these obstacles and elicit optimal immunity against CRC.

Stimulator of Interferon Genes (STING), an adaptor transmembrane protein localized in the endoplasmic reticulum, is a vital innate immune sensor that detects tumor-derived DNA^13^-^15^. The activation of the STING pathway induces a robust type I interferon (IFN) production, followed by activation of dendritic cells for the cross-priming of T cells, and elicitation of an adaptive immune response against tumors 15-17. Recent studies illustrated that STING is expressed in various human malignancies including melanomas, gastric cancer, and hepatocellular carcinoma, and it is correlated with T cell-mediated cancer immunity and the prognosis of those cancers 14, 18-20. Although the exact function of STING in human CRC has not been fully elucidated, the potential of STING in CRC has been strongly suggested in many animal studies, where it was found to mediate protection against CRC carcinogenesis 17, 21-23.

In this study, we aimed to explore the clinical value of STING as a prognostic immune biomarker in CRC patients and to evaluate its potential as an immunotherapeutic target in CRC.

## Materials and Methods

### Patients and tissue samples

This study was performed retrospectively on patients diagnosed with CRC at the CHA Bundang Medical Center (Seongnam, Korea) from 2002 to 2006. Tumor samples from 225 CRC patients were examined for STING and CD8 expression. The clinicopathological characteristics, such as gender, age, tumor location, differentiation, growth, stage, lymphovascular invasion (LVI), perineural invasion (PNI), microsatellite status (MSI), history of adjuvant therapy, recurrence, and survival outcome, were obtained from the electronic medical records at the institute. The 7^th^ edition of the American Joint Committee on Cancer guideline for tumor, node, and metastasis (TNM) classification was used for staging. The study was approved by the institutional review committee (IRB File No. 2017-11-054).

### Tissue microarray (TMA) construction and histologic analysis

Simple and precise paraffin TMAs were constructed using a conventional micro-compound table and a drill grinder. The original hematoxylin and eosin (H&E) slides were observed by pathologists. Two different tumor areas per case were selected for TMA construction. Core tissue biopsies, with a diameter of 3 mm, were taken from the individual paraffin blocks as donor blocks and arranged into tissue array blocks as recipient paraffin blocks using a trephine apparatus. All TMA blocks were stained with H&E for confirmation.

TMA blocks from CRC patients were cut into 5 μm-thick sections and immunohistochemical staining was performed using anti-STING (rabbit, clone D1V5L, Cell Signaling) or anti-CD8 (rabbit, clone SP57, Roche) antibodies. The BenchMark XT (Ventana) with heat-induced epitope retrieval (CC1 solution, Ventana) and the iView DAB detection kit (Ventana) was used as the visualization system. After the slides were mounted, high-resolution digital images of whole slides were taken with a BX43 microscope (Olympus). Immunofluorescent staining was performed on cryosectioned mouse tumor tissues with anti-CD31 (hamster, clone 2H8, Millipore) and anti-CD8 (rat, clone 53-6.7, BD Pharmingen) antibodies as previously described 8, 24. Immunofluorescent images were acquired with a LSM 880 confocal microscope (Zeiss).

Density measurement of STING^+^ or CD8^+^ areas was performed with the ImageJ software running the Fiji image processing package (https://imagej.net/Fiji). The color channels with hematoxylin and diaminobenzidine were separated and quantified to determine immunoreactive areas. Automated counting was applied for the analysis of all images. STING expression was assessed both in tumor and immune cells. The cut-off values to define high or low expression of STING or CD8 were the median values of all samples which were determined with ImageJ software.

### Mice and cell line

Male C57BL/6 mice (8 weeks old) were purchased from Orient Bio Inc. (Seongnam, Korea) and housed in a specific pathogen-free animal facility at CHA University (Seongnam, Korea). All experiments were approved by the Institutional Animal Care and Use Committee of CHA University (IACUC 170168). The MC38 murine colon cancer cell line was obtained from the National Cancer Center (Goyang, Korea). They were cultured in Dulbecco's Modified Eagle Medium supplemented with 10% fetal bovine serum (FBS) and 1% penicillin/streptomycin, and maintained at 37°C in a 5% CO_2_ incubator.

### Tumor model and treatment

All C57BL/6 mice were subcutaneously injected with 1 × 10^6^ MC38 cells on the right flank. When tumors reached > 5 mm in diameter, a STING agonist, 3'3'-cGAMP (10 μg in 50 μL of PBS; Invivogen), was intratumorally injected at D7, D10, and D13 after tumor implantation. Tumor volumes were measured using a digital caliper and calculated using the formula 1/2 × A × B^2^, where A is the longest diameter and B is its perpendicular diameter.

### Flow Cytometry Analysis

Tumors were harvested and chopped into several pieces. The tumor pieces were digested into single cell suspensions by incubating in digestion buffer [2 mg/mL collagenase D (Merck) and 40 μg/mL DNase I (Merck)] for 1 h at 37 °C. Cell suspensions were filtered using a 70-μm cell strainer (Corning) and a 40-μm nylon mesh to remove cell clumps. After washing with FACS buffer (1% FBS in PBS), cells were primed with antibodies targeting CD45 (30-F11, BD Pharmingen), CD4 (RM4-5, BD Pharmingen), CD8 (53-6.7, BD Pharmingen), CD3 (17A2, eBioscience), or ICOS (7E.17G9, eBioscience). Next, the cells were permeabilized using a permeabilization kit (eBioscience) and stained for IFN-γ (eBioscience). Data was acquired using a CytoFLEX flow cytometer (Beckman Coulter) and analyzed using the FlowJo software (Tree Star Inc., Ashland, OR, USA).

### Statistical analysis

Statistical analysis was performed using SPSS version 18.0 (IBM Corporation, Chicago, IL, USA). The correlation between STING expression and the clinicopathological variables was analyzed using the independent sample *t*-test for the continuous variables and the chi-square test for the discrete variables. In survival analysis, recurrence-free survival (RFS) was defined as the time interval between surgery and tumor recurrence or last follow-up. Overall survival (OS) was defined as the time interval from diagnosis to death or last follow-up. Kaplan-Meier method along with the log-rank test was used for survival analyses. The relationship between OS and the clinicopathological features was assessed using multivariate Cox proportional-hazards model. P < 0.05 was considered to be statistically significant.

## Results

### Baseline patient characteristics

The baseline clinicopathological characteristics are shown in Table 1. The male-to-female ratio was 1.25:1, and the mean age at diagnosis was 62 years. While 41% of the tumors were located in the rectum, the others were found in the colon. Most (85%) tumors were well- or moderately-differentiated tubular adenocarcinoma. LVI was present in 33% and PNI was present in 9% of the patients. All patients underwent surgical resection of the primary tumors. Distant metastasis was present in 8% of the patients at the time of diagnosis.

### STING expression and clinicopathological characteristics

**Figure 1:**
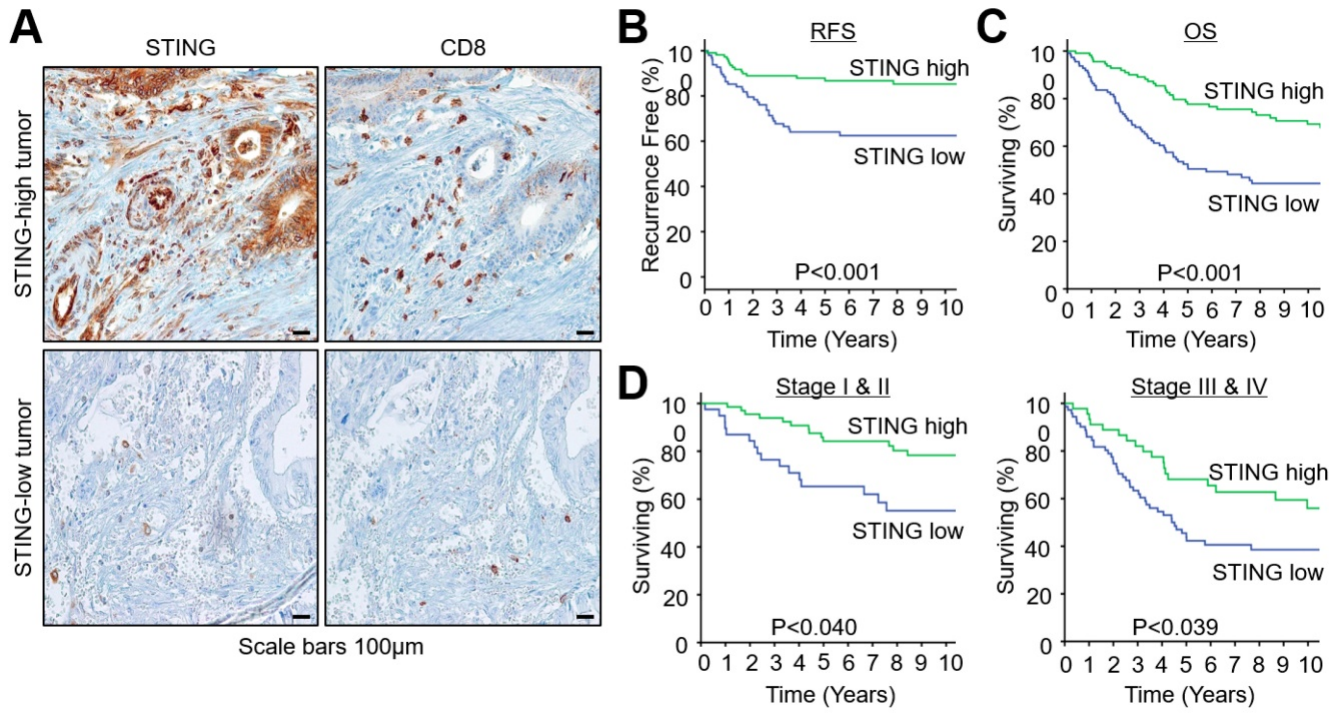
** STING expression and the prognosis of CRC according to STING expression.** (A) Representative images of STING and CD8 expression in CRCs. (B) Survival curves for recurrence-free survival (RFS). (C) Survival curves for overall survival (OS). (D) Survival curves for OS in early (stage I and II) and late (stage III and IV) stage tumors.

CRC tumor tissues were immunostained to visualize STING or CD8 expression (Figure 1A). Representative images of STING-high or -low tumors are shown in Figure 1A. The median of STING expression level in all samples was used as a cut-off value. STING is expressed in the cytoplasm of tumor cells, immune cells, and stromal cells. Notably, tumors with high STING expression had high intratumoral infiltration of CD8^+^ T cells.

Table 1 summarizes the correlation between clinicopathological characteristics and the level of STING expression. High STING expression significantly correlated with tumor stage (especially N) and intratumoral CD8^+^ T cells. No significant difference in STING expression was observed with respect to sex, age, tumor location, histology, growth type, PNI, MSI statue, and history of adjuvant therapy.

### STING expression and survival analysis

After a median follow-up of 74 months, 97 patients were found to have died at the time of survival analysis. The Kaplan-Meier survival analyses showed that patients with high STING expression had longer RFS when compared to those with low STING expression (5-year RFS rate, 85.3% vs. 62.5%; P < 0.001, Figure 1B). The same was also true for OS (5-year OS rate, 77.7% vs. 51.5%; P < 0.001, Figure 1C). When the OS of CRC patients was analyzed according to their stage, patients at the early stage (I + II) or advanced stage (III + IV) showed longer OS when they exhibited high STING expression (Figure 1D).

The clinicopathologic parameters significantly correlated with OS using univariate analyses were age (P = 0.047), T stage (P = 0.030), N stage (P < 0.001), CD8 expression (P = 0.009), STING expression (P < 0.001), presence of LVI (P < 0.001), and presence of PNI (P = 0.021) (Table 2). When multivariate Cox regression analysis was conducted with these variables, age (P = 0.009), STING expression (P = 0.012), and the presence of LVI remained as independent prognostic factors for OS in CRC patients (P = 0.001). Thus, it seems that high STING expression is a better prognostic factor than CD8 for OS in CRC patients both in univariate and multivariate analyses.

We also explored the potential of STING expression as a predictor for the response to chemotherapy in patients who received adjuvant chemotherapy. In these patients, patients with higher STING expression showed prolonged 5-year RFS rate (80.1% vs. 59.8%, P = 0.026) after chemotherapy compared with those with lower STING expression. Moreover, when we performed multivariate Cox regression analysis for RFS, high STING expression was indeed an independent predictive factor for chemotherapeutic efficacy (Table 3).

### Intratumoral STING treatment effectively suppresses colon cancer progression and enhanced intratumoral CD8^+^ effector T cells

**Figure 2:**
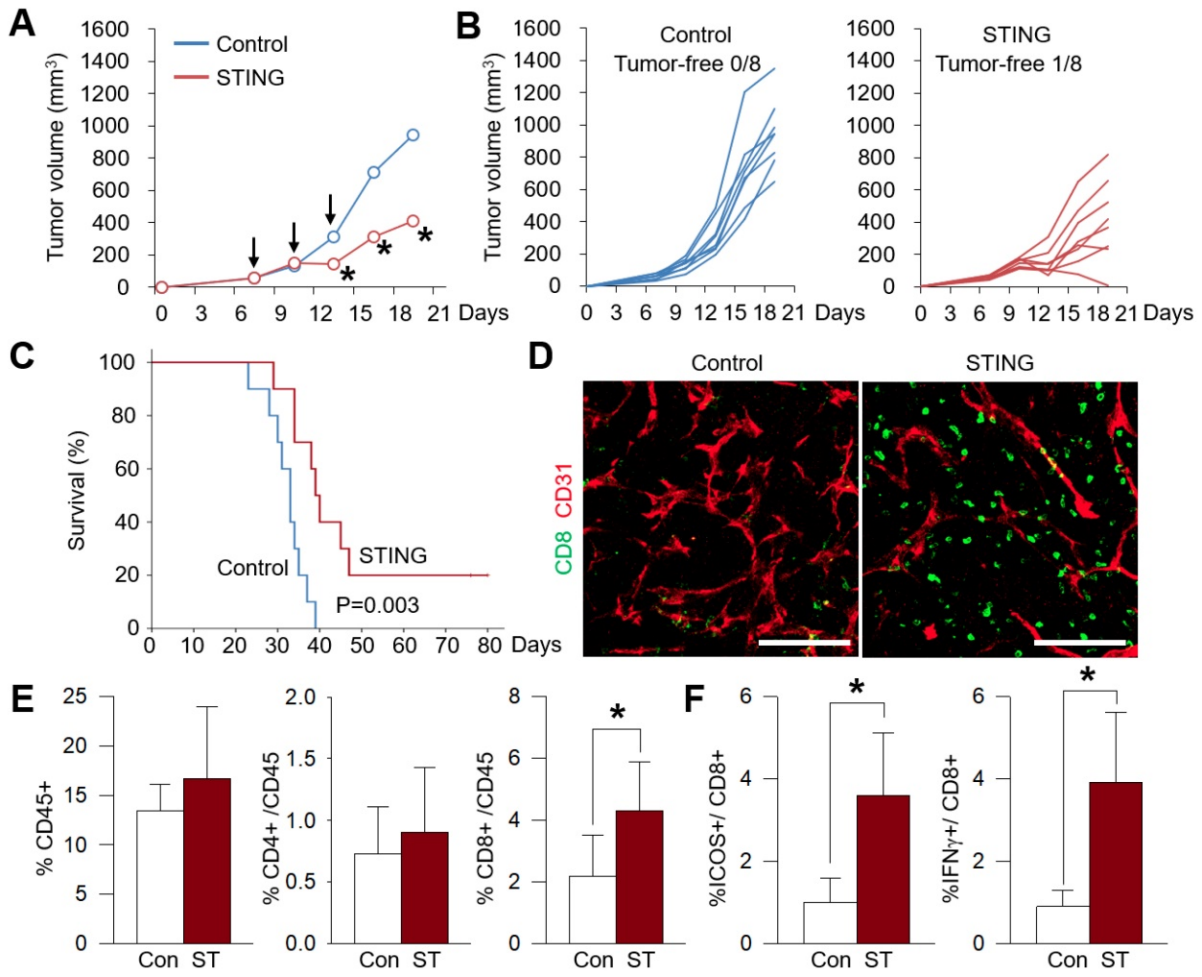
** Intratumoral STING activation effectively suppresses colon cancer growth and inflamed colon tumors with activated CD8^+^ T cells.** MC38 tumors were implanted subcutaneously into B57Cl/6 mice and treated with triple intratumoral injections of STING agonist, 3'3'-cGAMP (10 μg), when tumors reached >50 mm^3^. Each group, n = 8. Values are mean ± STD. *p < 0.05 versus control. Two-tailed Student's *t*-test was used. (A and B) Average (A) and individual (B) tumor growth curves. (C) Comparison of survival of tumor-bearing mice. (D) Representative images of tumor microenvironment showing CD8^+^ T cells (green) and CD31^+^ tumor blood vessels (red). Scale bars, 100 μm. (E) Comparison of CD45^+^, CD8^+^, or CD4^+^ cell within tumors. (F) Comparison of CD8^+^ICOS^+^, and CD8^+^GzB^+^ cells within tumors.

In order to confirm whether STING is a valid therapeutic target in CRC, we treated MC38 colon cancers with the STING agonist, 3'3'-cGAMP. Repeated intratumoral injections of the STING agonist suppressed MC38 tumor growth by 57% when compared to the control tumors (Figure 2A). Intriguingly, one STING-treated tumor completely regressed after the treatment (Figure 2B). Moreover, the survival of STING-treated mice was longer compared to the control mice (Figure 2C). Histologic analysis of the tumor microenvironment revealed an increase in CD8^+^ tumor-infiltrating T cells in STING-treated tumors (Figure 2D). When cell suspensions of the whole tumor were analyzed using flow cytometry, the CD8^+^ cytotoxic T cell fraction exhibited a two-fold increase when compared to control (Figure 2E). Moreover, ICOS and IFN-γ, which are markers for T cell activation, were also significantly upregulated in CD8^+^ T cells after intratumoral STING treatment, indicating the activation of intratumoral CD8^+^ T cells (Figure 2F). Collectively, intratumoral STING treatment was found to effectively suppress colon cancer progression through the enhanced intratumoral trafficking of activated CD8^+^ T cells.

## Discussion

STING signaling plays an important role in establishing anti-cancer immunity. Because DNA normally exists in the nucleus and mitochondria in mammalian cells, its presence in cytoplasm is a danger signal occurring in pathologic condition such as inflammation or cancer. STING is a cytosolic sensor that detects the presence of cytosolic DNA and triggers innate immune system and induces a potent adaptive immune response by upregulating type I IFN genes, enhancing CD8^+^ T cell cross-priming, and strengthening the anti-cancer effector function of T cells 15, 16, 25.

Our study elucidated that CRC patients with high STING expression in their tumors had longer survival rates and favorable prognosis compared to those with low STING expression. STING expression remained an independent prognostic biomarker for OS even after being adjusted for tumor stage and CD8 tumor-infiltrating lymphocytes (TILs). Our findings with CRC are in line with previous studies performed on other cancer types, such as hepatocellular carcinoma and gastric cancer 26, 27. Considering that CD8^+^ TILs are highly accumulated in STING-upregulated CRCs and STING is crucial for T-cell cross-priming and activation, intratumoral STING expression could be a useful immune biomarker in identifying T-cell-inflamed cancers.

In this study, we employed an immunogenic colon cancer model, MC38, which is a hypermutated and immunogenic cancer with mismatch repair deficiency to confirm the efficacy of STING-based immunotherapy 28. Correspondingly, this model showed a good response to STING-based immunotherapy, but the efficacy was not durable, and the rate of complete tumor regression was ~10%. This is because the activation of STING signaling could induce both favorable and unfavorable consequences during immune responses. At first, intratumoral STING activation elicits a strong type I IFN responses and activates CD8^+^ T cells to attack cancer cells. However, when these activated CD8^+^ T cells secretes IFN-γ, a potent anti-tumor effector cytokine, cancer cells upregulate PD-L1 as a defense mechanism to evade adaptive immune responses 29. Therefore, simultaneous blockade PD-1 or PD-L1 inhibitor could countervail STING-induced PD-L1 upregulation and enhance the efficacy of STING-based cancer immunotherapy 17. A few STING agonists, MK-1454 (Merck) and MIW815 (Norvatis), are currently undergoing phase I clinical trials in combination with anti-PD1 immune checkpoint inhibitors in advanced/metastatic solid tumors and lymphomas (NCT03172936, NCT03172936), respectively 30, 31. Preliminary results show encouraging therapeutic efficacy with acceptable toxicity levels, thereby supporting further clinical development of this novel combination immunotherapy 30.

Although STING is a meaningful prognostic biomarker for various solid tumors, it is still unclear whether the intratumoral STING expression levels are related to the response to immunotherapies. Therefore, the predictive value of STING expression needs further investigation in the ongoing clinical trials.

## Conclusion

In the present study, we revealed an independent prognostic role of STING signaling in CRC and demonstrated that STING-targeted cancer immunotherapy could remodel tumor immune microenvironment of CRC to enhance anti-cancer immunity.

## Figures and Tables

**Table 1:** Clinicopathological characteristics according to STING expression

Factors		All patients (n=225)	STING-low (n=112)	STING-high (n=113)	P-value
			N (%)	N (%)	
**Sex**	Female	100 (44.4)	52 (46.4)	48 (42.5)	0.551
	Male	125 (55.6)	60 (53.6)	65 (57.5)	
**Age (mean)**		62.0±12.1	61.6	61.6	0.976
**Location**	Right	36 (1.0)	19 (17.0)	17 (15.0)	0.520
	Transverse	14 (6.2)	7 (6.3)	7 (6.2)	
	Left	83 (36.9)	36 (32.1)	47 (41.6)	
	Rectum	92 (40.9)	50 (44.6)	42 (37.2)	
**Histology**	WD	14 (6.2)	7 (6.3)	7 (6.2)	0.267
	MD	177 (78.7)	91 (81.3)	86 (76.1)	
	PD	13 (5.8)	3 (2.7)	10 (8.8)	
	Mucinous	21 (9.3)	11 (9.8)	10 (8.8)	
**Growth**	Fungating	75 (33.3)	38 (33.9)	37 (32.7)	0.801
	Ulcerofungating	67 (29.8)	35 (31.3)	32 (28.3)	
	Ulceroinfiltrative	83 (36.9)	39 (34.8)	44 (38.9)	
**T**	T1	2 (0.9)	1 (0.9)	1 (0.9)	0.061
	T2	21 (9.3)	5 (4.5)	16 (14.2)	
	T3	162 (72.0)	82 (73.2)	80 (70.8)	
	T4	40 (17.8)	24 (21.4)	16 (14.2)	
**N**	N0	109 (48.4)	42 (37.5)	67 (59.3)	0.004
	N1	60 (26.7)	35 (31.3)	25 (22.1)	
	N2	56 (24.9)	35 (31.3)	21 (18.6)	
**Stage**	I	19 (8.4)	4 (3.6)	15 (13.3)	0.001
	II	88 (39.1)	36 (32.1)	52 (46.0)	
	III	100 (44.4)	58 (51.8)	42 (37.2)	
	IV	18 (8.0)	14 (12.5)	4 (3.5)	
**CD8**	Low	113 (50.2)	72 (64.3)	41 (36.3)	<0.001
	High	112 (49.8)	40 (35.7)	72 (63.7)	
**LVI**	No	151 (67.1)	67 (59.8)	84 (74.3)	0.020
	Yes	74 (32.9)	45 (40.2)	29 (25.7)	
**PNI**	No	204 (90.7)	102 (91.1)	102 (90.3)	0.835
	Yes	21 (9.3)	10 (8.9)	11 (9.7)	
**MSI**	Stable	137 (81.1)	63 (81.8)	74 (80.4)	0.621
	MSI-low	13 (7.7)	7 (9.1)	6 (6.5)	
	MSI-high	19 (11.2)	7 (9.1)	12 (13.0)	
**Adjuvant**	No	103 (45.8)	45 (43.7)	58 (56.3)	0.093
**Chemotherapy**	Yes	122 (54.2)	67 (54.9)	55 (45.1)	
**Adjuvant**	No	194 (86.2)	95 (84.8)	99 (87.6)	0.544
**Radiotherapy**	Yes	31 (13.8)	17 (15.2)	14 (12.4)	

WD, well differentiated; MD, moderatedly differentiated; PD, poorly differentiated; LVI, lymphovascular invastion; PNI, perineural invastion; MSI, Microsatellite Instability.

**Table 2:** Univariate and multivariate analyses for overall survival

Variables		Univariate	Multivariate	
		P-value	HR (95 CI)	P-value
Sex	(Male vs. Female)	0.875		
Age	(≥65 vs. <65)	0.047	1.733 (1.150-2.612)	0.009
Location	(Rectum vs. Colon)	0.567		
Histology	(WMD vs. Others)	0.333		
T	(T3,T4 vs. T1,T2)	0.030	1.670 (0.711-3.925)	0.239
N	(N1,N2 vs. N0)	<0.001	1.531 (0.981-2.391)	0.061
Distant metastasis	(M1 vs M0)	0.059		
CD8	(High vs. Low)	0.009	0.825 (0.536-1.270)	0.382
STING	(High vs. Low)	<0.001	0.573 (0.370-0.886)	0.012
LVI	(Yes vs. No)	<0.001	2.120 (1.345-3.339)	0.001
PNI	(Yes vs. No)	0.021	1.177 (0.645-2.149)	0.595
Adjuvant chemo	(Yes vs. No)	0.108		
Adjuvant radio	(Yes vs. No)	0.489		

HR, hazard ratio; CI, confidence interval

**Table 3:** Univariate and multivariate analyses for recurrence-free survival in patients who received the adjuvant chemotherapy.

Variables		Univariate	Multivariate	
		P-value	HR (95% CI)	P-value
Sex	(Male vs. Female)	0.979		
Age	(≥65 vs. <65)	0.147	2.463 (1.160-5.231)	0.019
Location	(Rectum vs. Colon)	0.241		
Histology	(WMD vs. Others)	0.684		
T	(T3,T4 vs. T1,T2)	0.206		
N	(N1,N2 vs. N0)	0.069	4.362 (1.013-18.788)	0.048
CD8	(High vs. Low)	0.353		
STING	(High vs. Low)	0.026	0.423 (0.197-0.908)	0.027
LVI	(Yes vs. No)	0.137	2.682 (1.259-6.714)	0.011
PNI	(Yes vs. No)	0.399		
